# A rare case of gastrobiliary fistula and gallbladder stones secondary to gastric foreign body: a case report

**DOI:** 10.3389/fmed.2026.1833620

**Published:** 2026-05-15

**Authors:** Zhirun Dong, Yaochen Wei, Jianguo Jia, Zhongying Li, Jinglong Sun, Shunxi Qu, Xiangming Ma

**Affiliations:** 1 Hebei North University, Zhangjiakou, Hebei, China; 2 Kailuan General Hospital, Tangshan, Hebei, China; 3 Department of Hepatobiliary Surgery, North China University of Science and Technology Affiliated Hospital, Tangshan, Tangshan, Hebei, China

**Keywords:** case report, cholelithiasis, gallstone, gastric foreign body, gastrobiliary fistula

## Abstract

Gastrobiliary fistula represents a rare and serious complication arising from various etiologies, including gallbladder calculi, peptic ulcer disease, neoplasms, and foreign bodies. Incidents of gastrobiliary fistula secondary to gastric foreign bodies are exceedingly uncommon. This report details the case of a 79-year-old woman who presented with intermittent upper abdominal pain persisting for over 10 months, with exacerbation noted in the preceding 3 days. Radiological evaluation revealed cholelithiasis with gas accumulation within the gallbladder, alongside linear hyperdense shadows suggestive of a potential gastrobiliary fistula. Intraoperative findings confirmed the presence of the fistula. The patient underwent successful cholecystectomy and fistula repair, resulting in an uneventful postoperative recovery and discharge. Given the rarity of gastrobiliary fistula, it is imperative that surgeons maintain a high index of suspicion and conduct thorough intraoperative exploration when preoperative imaging indicates possible fistulous communication. Importantly, the presence of a gastrobiliary fistula does not constitute a contraindication to laparoscopic surgical intervention.

## 1 Introduction

A fistula between the stomach and gallbladder represents an abnormal anatomical communication between these two organs, with gallstones identified as the predominant etiological factor (1, 2). The prevalence of gallbladder stones in the general population is estimated to be approximately 6% (3). Chronic irritation from gallstones leads to the gradual erosion of tissue, forming a firm adhesion between the gallbladder and stomach, ultimately resulting in fistula formation (2). Although gastrobiliary fistulas are rare and potentially life-threatening complications, their non-specific clinical manifestations often result in diagnosis being made intraoperatively in the majority of cases (92.1%) (4). This type of fistula is classified as an internal biliary fistula and can be further categorized based on the anatomical location of the fistulous tract, including gallbladder-duodenal, gallbladder-colonic, and other variants (5). Among these, the duodenum is the most frequently involved site (77–90%), followed by the colon (8–26.5%) and the stomach (2%) (4). The case under discussion is particularly unusual, as the fistula formed after a gastric foreign body perforated both the gastric wall and the gallbladder, thereby creating a gastrobiliary fistula. This abnormal communication subsequently facilitated the development of gallstones centered around the foreign body.

## 2 Case presentation

A 79-year-old woman patient reported experiencing intermittent upper abdominal pain for a duration exceeding 10 months. She was subsequently admitted to the hospital due to the exacerbation of abdominal pain persisting for 3 days, accompanied by jaundice affecting the skin and sclera, chills, and fever with a peak temperature of 38.5 °C. The patient had a medical history notable for hypertension over the past 5 years, with no other significant medical or surgical history, and no history of biliary interventions such as endoscopic retrograde cholangiopancreatography (ERCP). Additionally, there is a documented history of foreign body ingestion occurring more than 1 year prior. Physical examination identified tenderness localized to the upper abdomen, with no additional remarkable findings.

**Figure 1:**
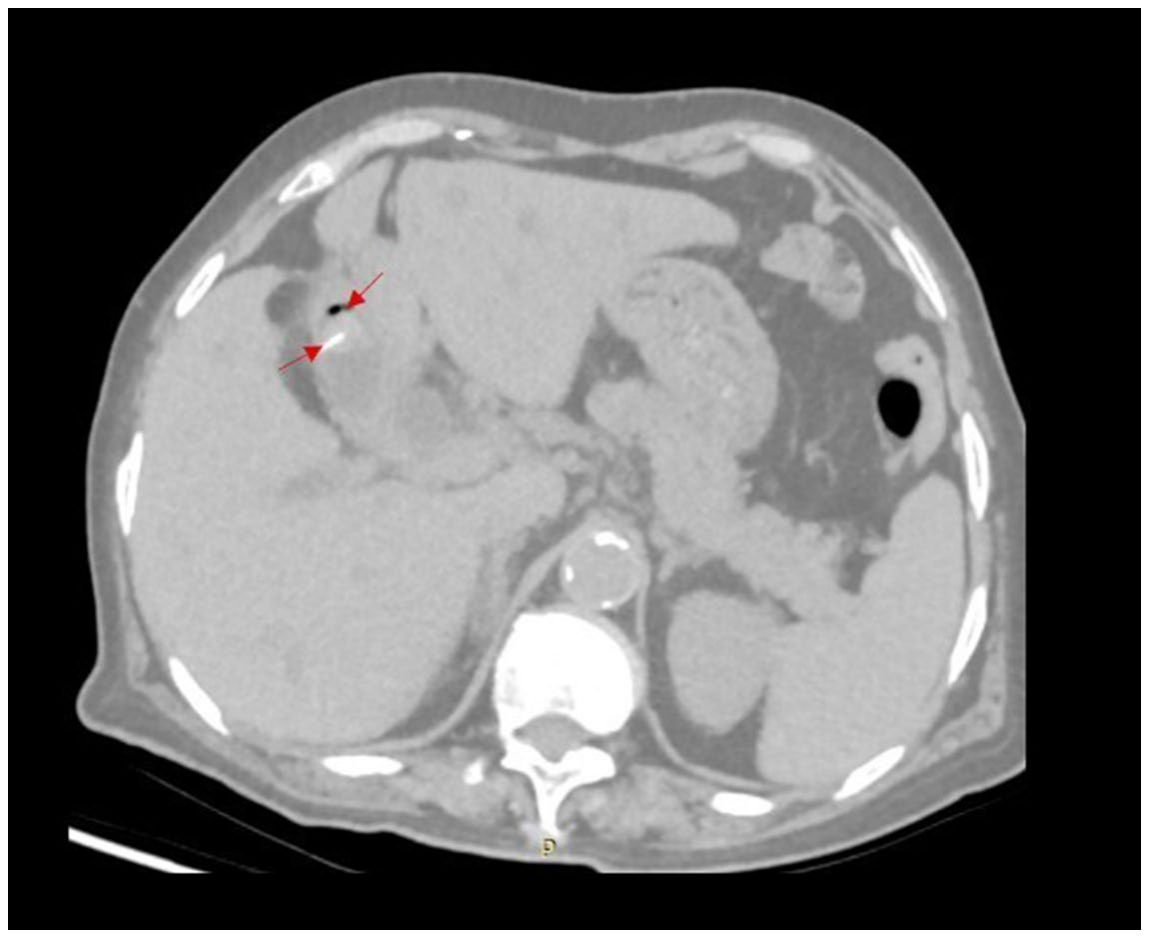
CT imaging reveals gas accumulation in the gallbladder and cord-like foreign bodies.

**Figure 2:**
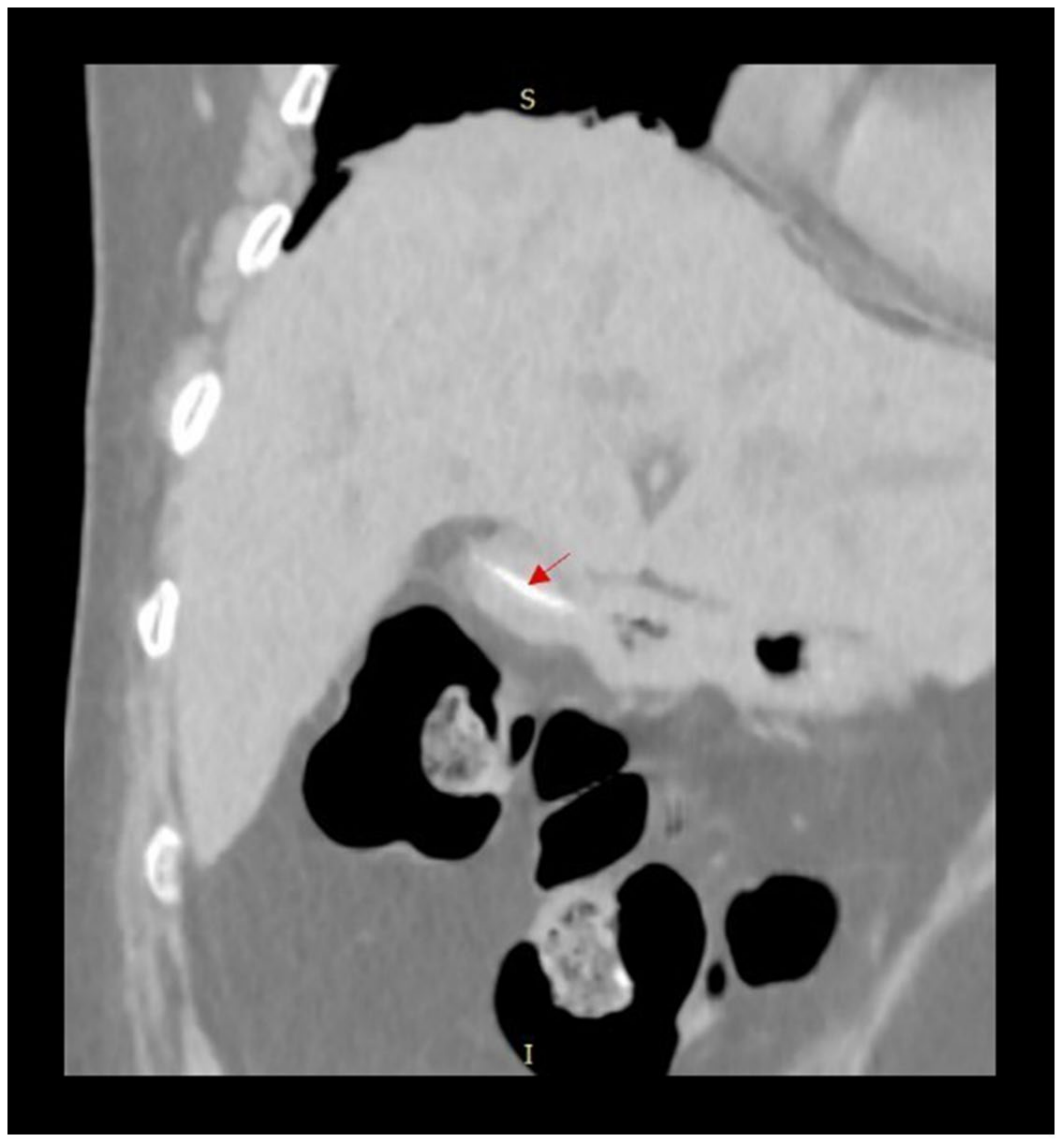
Imaging reveals a foreign object within the gallbladder, which is adjacent to the stomach.

Imaging findings upon admission demonstrated the presence of cholelithiasis accompanied by intraluminal gas within the gallbladder, as evidenced by linear hyperdense shadows observed on computed tomography (CT) imaging (Figure 1). The gallbladder was noted to be in close proximity to the stomach (Figure 2). Additionally, there was dilation of both intrahepatic and extrahepatic bile ducts, with calculi identified in the distal segment of the common bile duct. Magnetic Resonance Cholangiopancreatography (MRCP) reveals the presence of cholecystitis, cholelithiasis, calculi located in the distal portion of the common bile duct, and dilation of the biliary ducts. Laboratory evaluation revealed elevated serum levels of aspartate aminotransferase (AST) at 54 U/L, alanine aminotransferase (ALT) at 125 U/L, total bilirubin at 167.9 μmol/L, gamma-glutamyl transferase (GGT) at 438 U/L, and a white blood cell count of 7.7 × 10^9^/L.

Based on the patient’s imaging results, medical history, and laboratory evaluation, the diagnosis is gallstones and stones in the common bile duct. Preoperative assessment based on computed tomography (CT) imaging revealed high-density linear metallic-like shadows, radiographic evidence of gas accumulation within the gallbladder, and a close anatomical relationship between the gallbladder and the stomach. These findings raised suspicion for gallbladder foreign bodies and a potential gastrobiliary fistula. The patient had a confirmed diagnosis of cholelithiasis and choledocholithiasis. On 23 September 2025, the patient underwent a laparoscopic cholecystectomy, laparoscopic common bile duct exploration with stone extraction, and T-tube drainage. Intraoperative examination was concurrently conducted to verify the diagnosis of a gastrobiliary fistula. Intraoperatively, a fistulous connection was identified between the body of the gallbladder and the anterior wall of the proximal gastric antrum, thereby confirming the diagnosis of a gastrobiliary fistula. Surgical management included fistula repair, closing the terminal end of the gastric fistula using Prolene suture material, followed by complete cholecystectomy and common bile duct stone removal.

**Figure 3:**
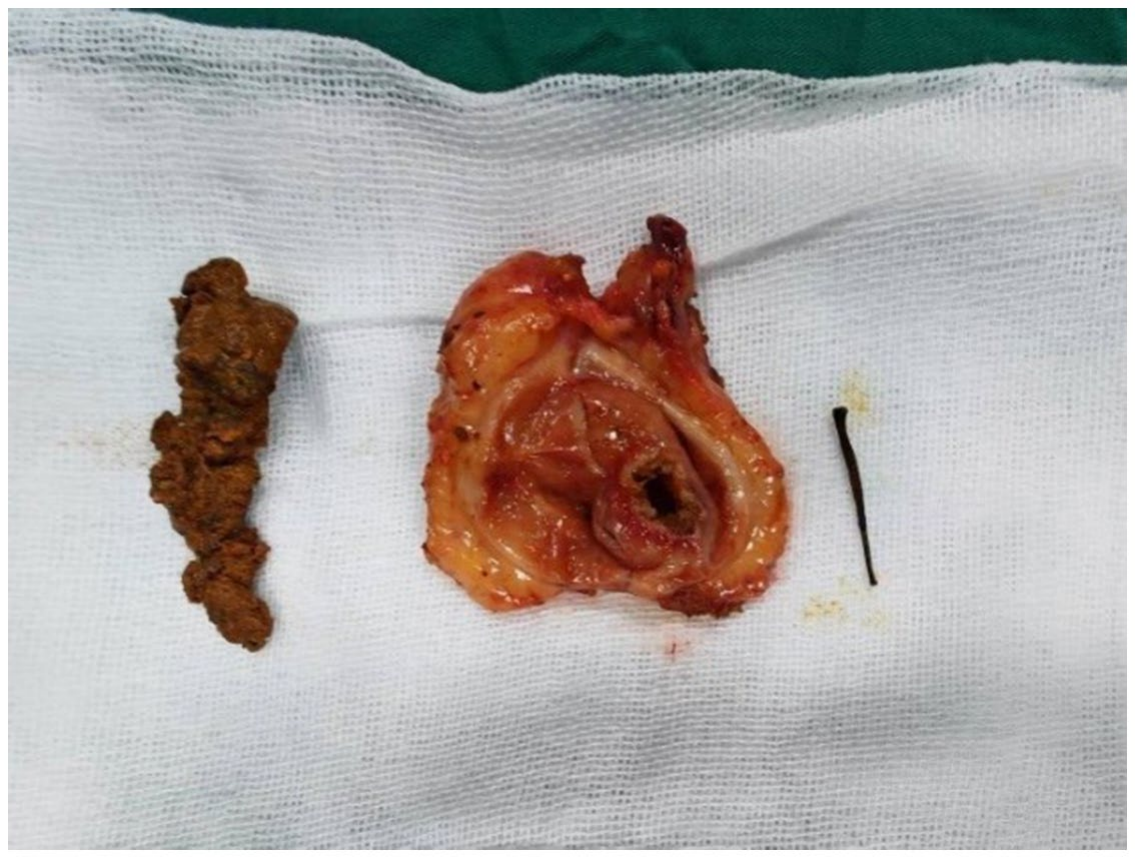
From left to right: soft stone, gallbladder and fistula opening, stone core.

**Figure 4:**
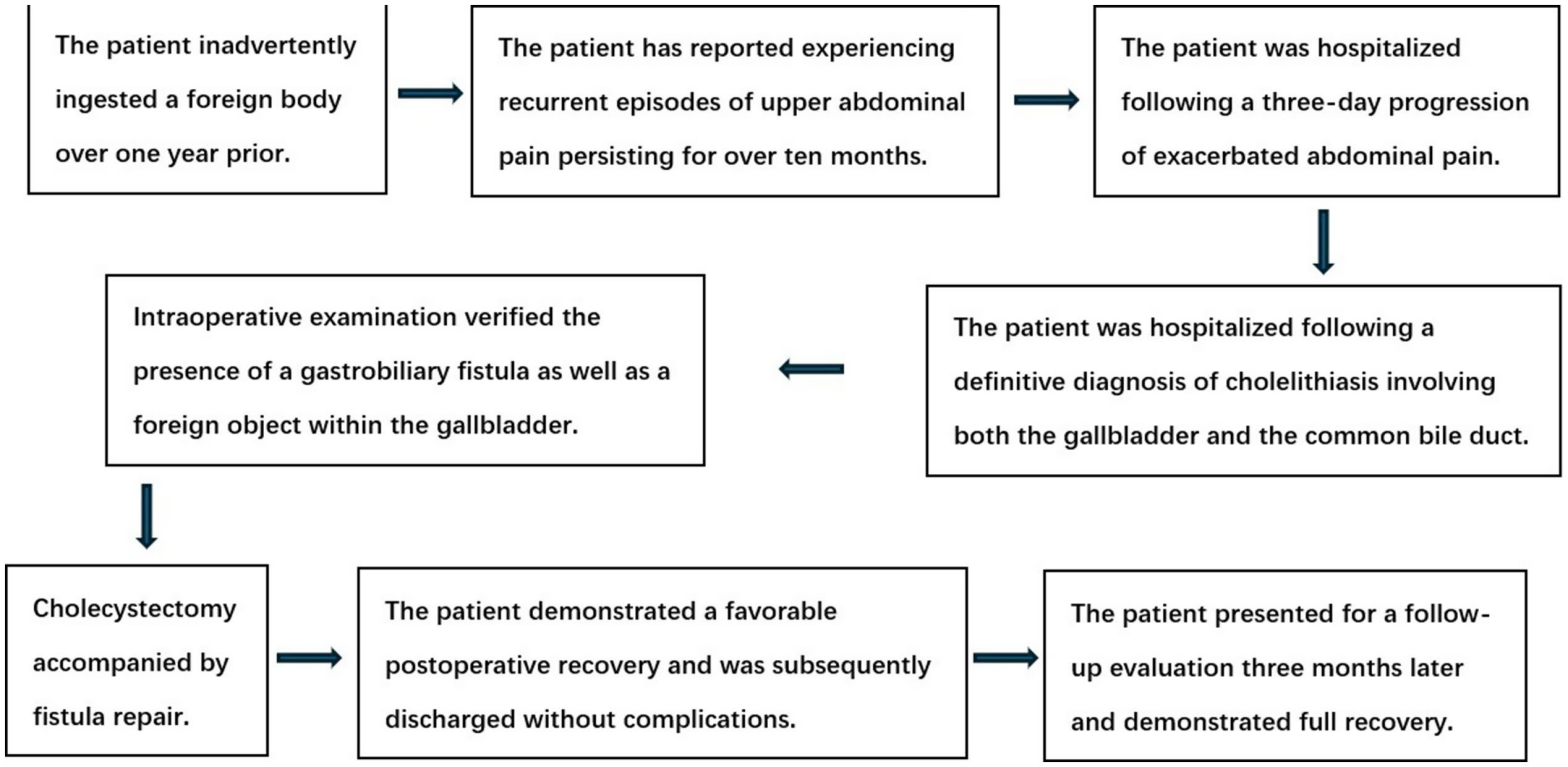
The clinical timeline.

Postoperative examination of the gallbladder identified a soft calculus. Upon separation, the calculus contained a metallic foreign object measuring approximately 2 cm in length and 1.5 mm in diameter, which is presumed to be the nucleus of the calculus (Figure 3). Postoperative abdominal computed tomography revealed characteristic alterations consistent with cholecystectomy, without any additional notable abnormalities. T-tube cholangiography confirmed the patency of the distal common bile duct, absence of residual calculi within the duct, and no evidence of contrast extravasation. Histopathological examination of the postoperative tissue specimen (Figure 3) demonstrated chronic cholecystitis accompanied by pseudopyloric gland metaplasia. On the second postoperative day, the patient ingested a limited quantity of water, followed by a small volume of liquid nourishment on the third postoperative day. The patient experienced an uneventful recovery following the surgical procedure and was subsequently discharged in good condition. Follow-up outcome: Three months after surgery, the patient returned to the hospital for a re-examination. After removal of the T-tube, cholangioscopy was performed through the sinus tract, revealing no obvious abnormalities. The patient reported no discomfort. The clinical timeline is shown in Figure 4.

## 3 Discussion

Emphysematous cholecystitis represents a highly severe variant of acute cholecystitis, distinguished by the accumulation of gas within the lumen and wall of the gallbladder. Furthermore, the presence of gas may also be observed in the biliary ducts or surrounding tissues (6). In this instance, the patient did not exhibit definitive indicators of systemic infection, such as significant fever or leukocytosis, at the time of admission. Computed tomography (CT) imaging identified the presence of gas and linear hyperdense shadows within the gallbladder. Following admission, the patient underwent symptomatic management comprising anti-inflammatory treatment, acid suppression, and fluid replacement, which resulted in clinical stabilization. Consequently, a diagnosis of emphysematous cholecystitis was not established. The patient exhibited no evidence of Courvoisier’s sign or progressive jaundice, nor was there a recent history of significant weight loss, neoplastic disease, or pertinent familial medical history. Laboratory investigations did not demonstrate elevated levels of tumor markers, including CA 19–9. Imaging studies, comprising computed tomography (CT) and magnetic resonance cholangiopancreatography (MRCP), revealed no findings suggestive of malignancy; consequently, additional contrast-enhanced CT imaging was deemed unnecessary. Subsequent surgical exploration and histopathological analysis postoperatively confirmed the absence of malignant pathology. Imaging studies revealed the presence of foreign bodies and gas accumulation within the gallbladder, which was located adjacent to the stomach (Figure 2). Consequently, a diagnosis of gastrobiliary fistula was strongly suspected. This diagnosis was subsequently confirmed through intraoperative exploration. Following the postoperative confirmation of foreign bodies within the gallbladder and the gastrobiliary fistula, the patient reported a history of accidental ingestion of foreign objects over 1 year prior, accompanied by several days of intermittent abdominal pain that resolved spontaneously without medical intervention. Given that the foreign body was confined solely to the patient’s gallbladder, and considering the presence of both gallbladder and common bile duct calculi with definitive surgical indications, a comprehensive multidisciplinary discussion was conducted. After fully incorporating the patient’s preferences, the decision was made to undertake intraoperative exploration of the gastrobiliary fistula concurrently with the surgical management of the gallbladder and common bile duct stones. This strategy facilitates simultaneous diagnosis and treatment, thereby obviating the need for preoperative upper gastrointestinal endoscopy.

This case report describes a gastrobiliary fistula resulting from the presence of a gastric foreign body. Specifically, a cord-like metallic object sequentially perforated the gastric wall and the gallbladder wall, ultimately entering the gallbladder. This process led to the formation of a gallstone centered around the foreign body. Fistulas are typically categorized according to the anatomical structures involved. The medical literature has documented nearly every possible anatomical combination; however, fistulas caused by gastric foreign bodies most frequently manifest as stomach-duodenum, stomach-jejunum, or stomach-colon fistulas. In contrast, occurrences of gastrobiliary fistulas are rarely reported (7–10). Frequent etiological factors contributing to the development of gastrobiliary fistulas encompass cholelithiasis, peptic ulcer disease, and neoplastic growths. Among these, cholelithiasis represents the predominant cause. In contrast, the occurrence of gastrobiliary fistulas resulting from gastric foreign bodies is exceedingly uncommon (1). The diagnosis of gastrointestinal fistulas, including gastrobiliary fistulas, primarily depends on imaging modalities and endoscopic evaluation. Initial diagnostic efforts should emphasize imaging methods, such as gastrointestinal contrast studies and computed tomography (CT) scans, as these techniques generally provide superior visualization of the presence and extent of fistulas compared to endoscopy (11). The computed tomography (CT) scan in this case distinctly reveals the presence of gas within the gallbladder (Figure 1). Fistulas associated with the gallbladder are frequently identified during surgical procedures. However, in this instance, preoperative imaging demonstrated unusual findings indicative of gas accumulation in the gallbladder. Consequently, careful intraoperative examination is imperative to detect the fistula, with focused attention on regions exhibiting dense adhesions and chronic inflammatory changes in the gallbladder. Additionally, intraoperative cholangiography may be utilized to more precisely delineate the fistula’s anatomical position (5). Existing studies have documented that the mortality rate associated with biliary-related fistulas ranges between approximately 8 and 13% (12, 13). Consequently, comprehensive preoperative assessment and preparation are imperative for patients presenting with gastrobiliary fistulas. The conventional management of gastrobiliary fistula entails performing a cholecystectomy in conjunction with fistula repair. Due to the advancement and widespread adoption of laparoscopic techniques, as well as the expertise of experienced surgeons, the existence of a fistula no longer constitutes a contraindication for laparoscopic intervention. However, the potential necessity to convert to an open surgical approach during the operation remains a possibility (5).

## 4 Conclusion

We present a rare case involving a gastrobiliary fistula and cholelithiasis secondary to the presence of a gastric foreign body. Imaging studies revealed gallstones accompanied by gas accumulation within the gallbladder, characterized by atypical linear high-density shadows. Careful intraoperative examination led to the identification of a gastrobiliary fistula. Subsequent postoperative analysis of the gallstones uncovered a cord-like metallic foreign object at the core of the calculi. The patient experienced an uneventful recovery and was discharged without complications. These findings underscore the importance of maintaining a high index of suspicion for fistula formation when atypical features, such as intracholecystic gas, are observed during preoperative assessment. In such cases, further diagnostic evaluation or thorough intraoperative exploration is warranted to mitigate the risk of severe adverse outcomes.

## Data Availability

The original contributions presented in the study are included in the article/supplementary material, further inquiries can be directed to the corresponding authors.
